# Composite Fish Collagen Peptide-Based Biopolymer Emulsion for Keratin Structure Stabilization and Hair Fiber Repair

**DOI:** 10.3390/polym17070907

**Published:** 2025-03-27

**Authors:** Wenwei Gu, Lei Gu, Ningping Tao, Xichang Wang, Changhua Xu

**Affiliations:** 1 College of Food Science & Technology, Shanghai Ocean University, Shanghai 201306, China; m220300894@st.shou.edu.cn (W.G.); leigu1023@163.com (L.G.); nptao@shou.edu.cn (N.T.); xcwang@shou.edu.cn (X.W.); 2 Shanghai Engineering Research Center of Aquatic-Product Processing & Preservation, Shanghai 201306, China; 3 Laboratory of Quality and Safety Risk Assessment for Aquatic Products on Storage and Preservation (Shanghai), Ministry of Agriculture, Shanghai 201306, China; 4 National R&D Branch Center for Freshwater Aquatic Products Processing Technology, Shanghai 201306, China

**Keywords:** collagen peptide, emulsion, secondary structure, heat stabilization, hair repair

## Abstract

Marine-derived proteins, rich in amino acids and bioactivity, serve as a natural and safe alternative to chemical haircare products. This study selected three highly bioactive fish-derived protein peptides and determined their optimal repair ratio using FTIR structural analysis and response surface methodology (RSM). A collagen peptide-based composite human hair repair emulsion (CHFRE) was formulated, and its repair efficacy on damaged hair (DH) was evaluated using scanning electron microscopy (SEM), Fourier transform infrared spectroscopy (FTIR), differential scanning calorimetry (DSC), and amino acid analysis. Following CHFRE treatment, the physical and chemical properties of damaged hair improved significantly. SEM analysis revealed enhanced hair luster, aligned cuticle scales, and a denser cortex. FTIR and DSC analyses showed a 5.94% increase in α-conformation content and a 28.44% rise in relative helical content (RHC), indicating enhanced protein stability and a conformation closer to that of normal hair. Additionally, the 14.63% increase in S=O transmittance suggested reduced oxidative damage. Amino acid analysis and hydrophobic amino acids, with specific increments of 16.77 g/100 g and 2.47 g/100 g, respectively, enhance hair affinity and keratin stability. This bio-based repair material effectively restores damaged hair structure, strengthens resistance to chemical damage, and ensures sustainability, safety, and biocompatibility, providing a promising approach for the development of natural hair repair products.

## 1. Introduction

Hair is one of the most fundamental and essential physiological features of mammals, serving to protect the skin, regulate temperature, and provide sensory functions. It plays a crucial role in these processes. The basic structure of the hair fiber is distributed in concentric layers and is divided into the cuticle, cortex, and medulla, which function mechanically as a whole. The cuticle primarily comprises β-keratin and inelastic proteins, forming a multilayered squamous structure made up of dead, flattened, overlapping cells. The outer surface of these squamous cells is coated with a lipoprotein layer approximately 10–14 nm thick, known as the epidermis [1]. Beneath the epidermis lies the outer cortex, which contains the majority of cystine residues. The innermost layer, the medulla, is located at the cortical interface, exhibiting variability in thickness and structure while having a low thionin content. The cells in the stratum corneum are interconnected by the cell membrane complex (CMC), which primarily consists of a lipid layer surrounding a central protein layer. The cortex constitutes the majority of the hair fiber mass (86–90%) and is primarily composed of keratin and structural lipids. It plays a crucial role in determining the hair’s appearance, strength, elasticity, and texture. Most of the hair pigment is concentrated in the cortex, particularly within melanosomes, where pigment granules contain melanin, the key determinant of hair color [2]. The medulla is a loosely vacuolated cellular structure located near the center of the hair fiber, becoming more prominent as the fiber diameter increases. It is rich in lipids, and its primary protein component is hyaluronan, which has relatively weak disulfide bonds [3]. Beyond natural aging [4], hair damage arises from hormonal imbalances, microbial overgrowth, environmental stressors (e.g., UV exposure or pollution), and cosmetic treatments (e.g., dyeing or bleaching). These factors degrade cuticle integrity, increase alkalinity, and cause keratin loss, often leading to hair breakage or loss [5,6,7,8]. Unfortunately, hair lacks the ability to self-repair. Given that hair issues profoundly impact individuals’ physical, psychological, and social well-being, hair repair and enhancement have become topics of significant interest [9]. As a result, the cosmetic industry is actively developing new haircare products aimed at improving hair health and appearance.

Natural materials (such as plants, animals, and microorganisms) are rich in protein resources and exhibit various biological activities (such as antihypertensive and antioxidant effects), while also possessing excellent biocompatibility, safety, and no side effects. Compared to chemical synthesis, peptide extraction from natural sources is more environmentally friendly and sustainable, aligning with the trends of green biotechnology development. In recent years, there has been a noticeable trend in the cosmetic industry toward incorporating marine-sourced ingredients into formulations due to their ability to meet diverse consumer demands and their broad range of bioactive properties, including antioxidant, moisturizing, and UV-resistant effects [10]. Marine-derived collagen hydrolysates are proteins and their hydrolysates are extracted from marine organisms, known for their excellent moisturizing properties and restorative effects [11]. Chen [12] investigated the efficacy of gelatin hydrolysate extracted from the skin of *Gadus macrocephalus* in reducing UV radiation-induced inflammation and collagen degradation. Gelatin and its hydrolysates from salmon skin have been shown to prevent photo-oxidation [13], while low doses of collagen hydrolysates enhance hydration and reduce transepidermal water loss. Additionally, hydrolyzed collagen derived from the fish bones of *Gadus macrocephalus* contains high levels of proline and hydroxyproline, which are essential for the stabilization of collagen’s triple-helix structure and the maintenance of protein integrity [14]. Meanwhile, the antioxidant peptide APTBP, obtained from tuna skeleton proteins, has been found to significantly inhibit lipid peroxidation in the linoleic acid emulsion system [15]. The hydrolysate of *Thunnus thynnus* head protein, A21, exhibits high antioxidant activity and remarkable functional properties [16]. Similarly, *Sardinella aurita* head protein undergoes enzymatic hydrolysis by proteases to yield antioxidant peptides, which serve as natural antioxidants to enhance the antioxidant properties of functional foods and prevent oxidative reactions during food processing [17]. Research has shown that marine-derived collagen peptides exhibit great potential in the development of modern haircare products. Natural collagen extracted from marine fish-processing by-products using acidic hydration technology, combined with ingredients such as hyaluronic acid and plant oils, has been developed into haircare products. The results demonstrate that it can effectively repair the hair cuticle [18]. Collagen extracted from salmon skin through hydrolysis and ultrafiltration also effectively repairs the hair cuticle and enhances shine [19]. Proteins and peptides contribute to hair repair primarily through two mechanisms: surface film formation and internal penetration. Surface film formation involves large proteins adsorbing to the hair surface via electrostatic interactions, creating a protective barrier. Internal penetration relies on small peptides diffusing into the cortex to stabilize keratin networks via disulfide bond rearrangement. Large-molecular-weight proteins and peptides predominantly adhere to the surface of damaged hair via electrostatic adsorption or chemical cross-linking, forming a protective protein film that shields the hair from adverse external conditions [20]. In contrast, small-molecular-weight proteins and peptides possess superior permeability, allowing them to penetrate the lipid layer on the hair fiber’s surface and reach the interior of the hair strand. By facilitating the rearrangement of internal chemical bonds, these peptides improve hair texture and overall structural integrity [21]. Thus, we selected three fish protein peptides, *Takifugu obscurus* skin peptides (TSPs), *Pneumatophorus japonicus* bone peptides (PBPs), and *Pneumatophorus japonicus* head peptides (PHPs), with good biological activities to verify their combined (TSP–PBP–PHP) repair potential.

Currently available peptide delivery systems include emulsions, micelles, and peptide-based nanocarriers. Micelles possess an amphiphilic structure with both hydrophilic and hydrophobic domains, allowing them to effectively encapsulate and transport small bioactive molecules, thereby facilitating deeper penetration into the hair shaft [22]. However, micellar systems often require high concentrations of surfactants [23,24], which may lead to cuticle damage or scalp irritation when used excessively. Peptide-based nanocarriers, owing to their nanoscale dimensions, exhibit enhanced penetration capabilities and can form a sustained-release system on the hair [25] or skin surface [26], thereby providing prolonged repair effects. However, these technologies typically involve complex fabrication processes and high production costs, limiting their large-scale application in commercial haircare products. In contrast, emulsions offer a more cost-effective and scalable natural repair solution, making them suitable for the development of commercial haircare formulations [27]. Emulsions are widely utilized in hair and skincare products [28,29], as they can be optimized through emulsifiers and thickeners to improve permeability and adhesion, ensuring more effective delivery of active ingredients to damaged hair [30]. However, conventional emulsions primarily remain on the hair surface, making deep repair more challenging. The innovation of this study lies in the development of a composite fish collagen peptide emulsion (CHFRE), which optimizes the ratio of the three bioactive peptides. This formulation not only forms a protective film on the hair surface but also enhances the penetration of small-peptide molecules, thereby improving the repair efficacy within the hair structure.

Consequently, the design and development of novel protein-based, eco-friendly, and sustainable products for human hair restoration hold significant potential for future advancements. In this study, we aim to validate the hair fiber repair effects of the selected fish-derived collagen peptide formulations and enhance their practical application potential by incorporating them into an emulsion system.

## 2. Materials and Methods

### 2.1. Materials and Reagents

*Takifugu obscurus* skin processing by-products, *Pneumatophorus japonicus* head processing by-products, and *Pneumatophorus japonicus* bone processing by-products, provided by Zhejiang Xingye Group Company Limited (Zhoushan, China), were stored frozen at −20 °C. Complex proteases were obtained via the fermentation of *Bacillus amyloliquefaciens* and *Bacillus licheniformis* (Novozymes, Bagsværd, Denmark), alkaline protease (Macklin, Shanghai, China), papain (Macklin), and trypsin (Macklin); Ceteartrimonium Chloride (Lemay Chemical, Guangzhou, China), CETEARYLALCOHOL (Macklin), EDTA-2Na (Bioss, Boston, MA, USA), CL12490 (Lemay Chemical, Guangzhou, China), CL11680 (Lemay Chemical, Guangzhou, China), and Citric Acid (Macklin); and concentrated sulfuric acid (Collins, Shanghai, China).

### 2.2. Sample Pre-Treatment

#### 2.2.1. Preparation of TSP, PBP, and PHP

The skin of *Takifugu obscurus* was cut into pieces (approximately 1 cm × 1 cm), and the bones and heads of *Pneumatophorus japonicus* were crushed until no visible particles remained. Then, 100 g portions were packed into bags and stored in a refrigerator at −40 °C for later use.

The skin of *Takifugu obscurus* was extracted with distilled water at a skin-to-water ratio of 1:3 (*w*/*v*) in a water bath for 3 h at 90 °C. Based on the optimal conditions determined from preliminary laboratory tests, 2% of the complex protease (obtained by the fermentation of *Bacillus amyloliquefaciens* and *Bacillus licheniformis*) was added, and the enzyme was digested by shaking in a water bath at a temperature of 55 °C and a pH of 7.0 for 2 h. After the enzyme digestion, the enzyme was inactivated by heating for 15 min at 95 °C and then centrifuged at 8000 r/min for 15 min at 4 °C. The digest was cooled to room temperature and then centrifuged at 8000 r/min for 15 min at 4 °C. The supernatant was collected in a 500 mL conical flask, and 1.5% activated carbon was added to decolorize the peptide for 30 min at 60 °C and then filtered to obtain the digest of the peptide of *Takifugu obscurus* skin. The digest was dried in the vacuum freeze dryer (FD-80, BICOOL, Beijing, China) for 48 h to remove water, and then the peptide powder was obtained.

The heads of *pneumatophorus japonicus* were minced, and distilled water was added at a head-to-water ratio of 1:4 (*w*/*v*). Then, 0.32% of compound protease (a mixture of alkaline protease and trypsin at an enzyme activity ratio of 1:3) was added, and the enzymatic digestion was carried out for 4 h at 50 °C with shaking at 120 rpm in a water bath. The enzymes were then inactivated at 90 °C for 15 min. After cooling to room temperature, the digest was centrifuged at 8000 r/min for 15 min at 4 °C. The supernatant was collected in a 500 mL conical flask, decolorized with 1% activated carbon in a water bath for 30 min at 30 °C, and then filtered to obtain the enzyme digest of *Pneumatophorus japonicus* heads peptide. The digest was dried in a vacuum freeze-dryer for 48 h to remove the water, and then the *Pneumatophorus japonicus* heads peptide powder was obtained.

The bones of *Pneumatophorus japonicus* were minced, and distilled water was added at a bones-to-water ratio of 1:3.62 (*w*/*v*). The total enzyme dosage of papain and trypsin was 5000 U/g, calculated based on the weight of mackerel bones and the enzyme activity of the two enzymes. The enzymatic digestion was carried out for 4 h at 62 °C and pH 7.0 with shaking at 120 rpm in a water bath, followed by enzyme inactivation at 95 °C for 15 min. After cooling to room temperature, the digest was centrifuged at 8000 r/min for 15 min at 4 °C. The supernatant was collected in a 500 mL conical flask, and 1.5% activated carbon was added to decolorize the enzyme solution for 30 min at 60 °C and then filtered to obtain the enzyme digest of the peptide of *Pneumatophorus japonicus* bones. The enzyme digest was dried in a vacuum freeze-dryer for 48 h to remove the water, and then the *Pneumatophorus japonicus* bone protein peptide powder was obtained.

#### 2.2.2. NH, DH Sample Collection and Preparation

Normal hair (NH) samples were collected from ten female volunteers (aged 20–28 years) at Shanghai Ocean University. All participants had naturally healthy hair, with no history of cosmetic treatments such as bleaching, coloring, or perming, and exhibited no signs of frontal hairline recession.

Damaged hair (DH) samples were prepared by subjecting the NH samples to bleaching with a commercial hair bleach for 1.5 h, followed by dyeing with a hair dye for 1 h. Subsequently, the samples were thoroughly rinsed with ultrapure water and air-dried at room temperature. A comparison of the images of DH and NH samples is shown in Figure S6.

#### 2.2.3. Hair Sample Allocation and Initial Treatment

Normal and damaged hair samples were washed three times with ultrapure water under oscillation for 1 min each time to remove surface impurities, then naturally air-dried. A total of 29 sample groups were designed in the experiment, including one group of NH and one group of DH. The remaining 27 groups were repair treatment groups based on DH, specifically comprising 9 groups treated with varying ratios of TSP and PBP, 9 groups with different ratios of TSP and PHP, and another 9 groups with different ratios of PBP and PHP. Following treatment with the aforementioned CHFREs, the hair samples were rinsed with ultrapure water under oscillation, naturally air-dried, and stored for further analysis. After treatment, the hair fibers were carefully removed with tweezers, washed three times, and left to dry naturally at room temperature for 12 h. The samples were sequentially packed and stored in a numbered order. Three samples of normal hair and three samples of damaged hair were selected for subsequent analysis.

### 2.3. Screening of the Optimal Ratio of Three Fish Collagen Peptides

#### 2.3.1. Infrared Spectral Acquisition

Infrared spectral data were collected using the ATR accessory of a Fourier transform infrared (FTIR) fiber optic imaging system (Spotlight 400, PerkinElmer, Waltham, MA, USA). The hair sample was placed directly over the ATR crystal, ensuring full coverage, and the obtained spectra were subsequently processed accordingly. The spectra were acquired using the following parameters: a wavenumber range of 4000–600 cm^−1^, a spectral resolution of 4 cm^−1^, and scanning signal accumulation of 16 times. Baseline correction and average spectrum calculation of the acquired protein infrared and transmittance spectra were performed using PerkinElmer Spectrum 10.4.3. The second-order derivative IR spectra were then smoothed by 13-point Savitzky-Golay fitting. After Fourier deconvolution of the amide I band using PeakFit 4.12 and second-order derivative peak fitting with a Gaussian function, the stable minimum residuals were obtained. The relative percentage content of the corresponding secondary protein structures was calculated based on the integrated area of each subpeak.

#### 2.3.2. One-Factor Experimental Design

A: Effect of TSP fraction on hair α-conformation and transmittance of S=O at 1042 cm^−1^; B: Effect of PBP fraction on hair α-conformation and transmittance of S=O at 1042 cm^−1^; C: Effect of PHP fraction on hair α-conformation and transmittance of S=O at 1042 cm^−1^. Two comparisons were made between A, B, and C to check each other.

#### 2.3.3. Response Surface Design

According to the laboratory pre-study, a protein peptide concentration of 6 mg/mL(*w*/*v*) was found to be optimal. The 6 mg was divided into ten parts, e.g., TSP–PBP = 1:9 (the peptide mixtures were prepared at mass-to-mass ratios, *w/w*), whereby one part of TSP and nine parts of PBP were added. The final calculation was 0.6 mg of TSP and 5.4 mg of PBP added to 10 mL of the protein solution, with TSP noted as “number 1” and PBP noted as “number 9”.

Based on the one-way experiment, α-conformation (Y1) and transmittance at the characteristic S=O peak at 1042 cm^−1^ (Y2) were selected as the response variables. Three independent variables were considered: the number of TSP copies (A), the number of PBP copies (B), and the number of PHP copies (C). A three-factor, three-level response surface design was implemented using the Box–Behnken method with an 11-center combination in Design-Expert 11 software for optimization. The data were fitted and analyzed using Design-Expert 11, and the analyzed factors and levels are presented in Table 1.

### 2.4. Formulation Development of Fish-Derived Protein Peptide Haircare Film and Evaluation of Its Haircare Effect

#### 2.4.1. Determination of Particle Size and Zeta Potential of Lyophilized TSP, PBP, and PHP Powders

The dry powder samples of TSP, PBP, and PHP were diluted to 1 mg/mL with deionized water. After equilibrating for 30 min, the zeta potential and particle size of the samples were measured using a Malvern Zetasizer Pro Dynamic Light Scattering Particle Sizer (Zetasizer Pro, Malvern Panalytical, Malvern, UK) at room temperature.

#### 2.4.2. Hair Treatment with Different CHFRE Concentrations

The NH and DH samples were thoroughly rinsed three times with ultrapure water, each for 1 min, to remove surface impurities. After rinsing, the hair samples were naturally air-dried at room temperature. The DH samples were evenly cut into seven groups: one group was used as a negative control (blank treatment), and the remaining six groups were treated with CHFREs containing different concentrations (0%, 1%, 2%, 3%, 4%, and 5%) of TSP–PBP–PHP for 1 h. The NH samples were used as a positive control group. After the treatment, the hair samples were rinsed three times with ultrapure water, air-dried naturally at room temperature for 12 h, and then sequentially numbered for storage and future use.

#### 2.4.3. Optical Microscope Observation of Hair Strands After CHFRE Treatment

Hair samples from each group were cut to approximately 5 cm and fixed on slides, including both healthy and unhealthy samples. The appearance and morphology of the original hair, as well as hair samples treated with different CHFREs, were observed. Additionally, the surface gloss of the hair fibers was observed using an orthogonal metallurgical microscope (ML8000, Mezis, Shanghai, China) equipped with a 10× eyepiece and a 40× objective lens to obtain the microscopic images of the hair samples.

#### 2.4.4. SEM of Hair After CHFRE Treatment

Each group of hair samples was cut to a length of about 1 cm. The hair samples were gently clamped with tweezers, adhered to the carrier stage using conductive adhesive, and sprayed with gold for 50 s. The samples were then examined using a scanning electron microscope (SUS5000, Hitachi, Tokyo, Japan) at an accelerating voltage of 5 kV, with magnifications of 500×, 1000×, and 2000×, to obtain SEM images of the hair samples.

#### 2.4.5. Infrared Spectral Acquisition of Hair After CHFRE Treatment

The experimental manipulation is consistent with Section 2.3.1.

#### 2.4.6. Differential Scanning Calorimetry Analysis of Hair After CHFRE Treatment

The hair samples of each group were placed in an environment of 25 °C and 65% relative humidity for 48 h. After equilibration, the hair was cut into pieces smaller than 1 mm in length. Accurately weighed hair samples (5–6 mg) were placed on an aluminum plate with a nitrogen flux rate of 50 mL/min during the operation and detected using a differential scanning calorimeter (Q2000, TA Instruments, New Castle, DE, USA).

The program was set up as follows: first, the samples were heated from room temperature to 60 °C and maintained for 30 min to remove surface-free water. Then, they were cooled down to 30 °C at a rate of 10 °C/min and subsequently heated up to 270 °C at the same rate, while absorption curves were recorded. Each hair sample was measured in triplicate, and the average peak absorption temperature and average peak absorption area were calculated. The relative helix content (RHC) of hair keratin was determined using the following equation:

$$RHC=\frac{{\Delta H}_{d}}{{\Delta H}_{d0}}\times 100\%$$

ΔH_d_ is the enthalpy of denaturation (J/g) and ΔH_d0_ is the enthalpy of denaturation of damaged hair. The peak temperature (T_d_) represents the denaturation temperature of the α-helix peak (°C).

#### 2.4.7. Determination of Amino Acid Content of Hair After CHFRE Treatment

Each of the eight groups of hair samples was cut into 5 mm pieces, and 20 mg of hair was accurately weighed into a hydrolysis tube. Then, 10 mL of 6 mol/L hydrochloric acid was added, and the mixture was cooled in a refrigerator at 4 °C for 5 min before being vacuum-sealed. The sealed tube was then hydrolyzed in an oven at 110 °C for 22 h. After hydrolysis, the sample was removed from the oven, cooled to room temperature, and filtered through filter paper into a 50 mL volumetric flask. The hydrolysis residue was repeatedly washed with small amounts of distilled water, followed by volume adjustment and thorough mixing. The hydrolysis tube was also rinsed with distilled water several times to ensure complete transfer of the hydrolyzed solution, after which the final volume was fixed and mixed well. We then pipetted 1 mL of filtrate into a 25 mL test tube and dried it under vacuum at 50 °C. The residue was dissolved in 1 mL of distilled water and vacuum drying was repeated until complete evaporation. Next, 2 mL of pH 2.2 sodium citrate solution was added to the test tube to fully dissolve the residue. After shaking and mixing, the solution was filtered using a 0.22 μm aqueous filter membrane, and the filtrate was collected in a 2 mL injection vial. The sample was then stored in a refrigerator at −20 °C for further analysis using an amino acid autoanalyzer (LA-8080, HITACHI, Tokyo, Japan). After instrumental analysis and subsequent calculations, the amino acid composition of each hair sample group was determined.

### 2.5. Statistical Analysis

The data were analyzed for the comparative significance of differences using SPSS 27.0 software using Duncan’s letter marking method for multiple comparisons. The method ranks individual treatment means in ascending order. The highest mean is assigned the letter ’a’ if it differs significantly from lower means, which are progressively labeled with subsequent letters (e.g., ’b’ and ’c’). When the level of significance was 0.05, a lowercase Latin letter a was marked after the largest mean until a mean that differed significantly from it was marked with the letter b. The method was repeated until the smallest mean was marked with the letter b. This was repeated until the smallest mean was labeled and compared. Response surface analysis methods were optimized and data were fitted using Box–Behnken11 in Design-Expert software 13.0. The obtained infrared spectrograms of proteins were corrected and calculated using PerkinElmer Spectrum 10.4.3. The smoothed, processed second-order derivative IR spectrograms were then fitted by Savitzky–Golay. The relative percentage content of the corresponding secondary structure of the protein obtained was calculated using PeakFit 4.12. Images were generated using Origin 2021 and GraphPad Prism version 9.0 software.

## 3. Results and Discussion

### 3.1. Infrared Spectral Analysis of Three Fish-Derived Keratin Repair Peptides in Different Ratios

Infrared spectroscopy (Figure S7) and amino acid analysis (Table S4) indicate that TSP is characterized by a low lipid content, a high proportion of short peptides, and a rich amino acid profile, suggesting its potential suitability for deep repair. PBP contains a higher lipid content, which may facilitate the formation of a stable emulsified structure within the emulsion system. PHP exhibits greater protein integrity, which may contribute to film formation and enhance hair strength. Based on the laboratory pre-study, TSP demonstrated a significant hair repair effect [31]. Considering that the effect of the three protein peptides on the hair on hair may vary, a further investigation was conducted to optimize their ratio and determine the most effective combination for hair repair.

Protein secondary structure changes directly affect keratin stability, which can be assessed through structural analysis. In damaged hair, alterations in protein secondary structure are commonly observed. The α-helix [32,33] is one of the predominant secondary structures in hair, playing a crucial role in its physical properties. However, exposure to chemical or physical damage can lead to a reduction or disruption of the α-helix, resulting in decreased elasticity and strength. Although β-folding [34] is less abundant, their presence may increase in damaged hair, leading to changes in its physical properties of the hair. Such structural changes are caused by the constant mechanical and chemical stimulation of protein molecules in the hair. These stimuli can disrupt existing intermolecular hydrogen bonds and promote the formation of new ones, thus affecting the secondary structure in the hair fiber.

As shown in Figure S1, the infrared second-order derivative spectrograms of the treated DH in the protein band became significantly smoother and more similar to the NH at TSP–PBP = 7:3, 8:2, and 9:1.Additionally, Table 2 indicates that the proportion of α-conformations (α-helix and random coil) exceeded 30% at TSP–PBP ratios of 3:7, 4:6, 5:5, 6:4, 7:3, 8:2, and 9:1, suggesting an improved restorative effect on hair within this range.

As shown in Figure S2, the infrared second-order derivative spectrograms of the treated DH in the protein band became significantly flatter and more similar to the NH at TSP–PHP = 5:5, 6:4, and 9:1. Additionally, Table 3 indicates that the proportion of the α-configuration (α-helix and random coil) exceeded 30% at TSP–PHP ratios of 3:7, 4:6, 5:5, and 6:4, suggesting a better restorative effect on hair within this range.

As shown in Figure S3, the infrared second-order derivative spectrograms of treated DH in the protein band became significantly flatter and closer to the NH at PBP–PHP = 5:5, 6:4, and 9:1. Additionally, Table 4 shows that the proportion of α-conformations (α-helix and random coil) exceeded 30% at PBP–PHP ratios of 3:7, 4:6, 6:4, and 7:3, suggesting that this range has a better restorative effect on hair.

Due to the large relative atomic mass of sulfur atoms, it is often difficult for them to directly produce significant absorption peaks in infrared spectra. As a result, the presence and disruption of disulfide bonds, particularly the formation of sulfenic or sulfonic groups (typically caused by disulfide bond oxidation) are of great significance in assessing oxidative hair damage. These structural changes are often indirectly reflected by the characteristic S=O peak near 1042 cm^−1^ [35]. In general, a sharper and more intense S=O peak at 1042 cm^−1^ indicates more severe oxidative damage. In the infrared spectra of normal hair, the characteristic S=O peak is almost undetectable, and the spectral line appears relatively smooth (Figure S4). In contrast, damaged hair exhibits a significantly enhanced S=O signal, confirming more severe oxidative damage. Since transmittance is the reciprocal of absorbance, visualizing the transmittance data provides a clearer comparison of oxidative damage at the characteristic S=O peak near 1042 cm^−1^. Specifically, higher transmittance indicates less oxidative damage, whereas lower transmittance suggests more severe damage.

NH exhibited an S=O transmittance of 55.05% at 1042 cm^−1^. The assessment of DH repair should not rely solely on the IR spectra in the amide I band (1662–1658 cm^−1^) and amide II band (1650–1648 cm^−1^) regions but should also consider the extent of oxidative damage repair. Figure S4 shows that different ratios of TSP–PBP, TSP–PHP, and PBP–PHP effectively mitigate oxidative damage. At 1042 cm^−1^, the peak shape of treated DH becomes flatter, indicating reduced oxidative stress. Table 5 confirms that the following ratios showed the best effects against oxidative damage: TSP–PBP = 3:7, 4:6, 5:5, 6:4, 7:3, 8:2, and 9:1; TSP–PHP = 6:4, 7:3, and 8:2; and PBP–PHP = 4:6, 5:5, 6:4, 7:3, 8:2, and 9:1.

### 3.2. One-Factor Experimental Analysis

**Figure 1:**
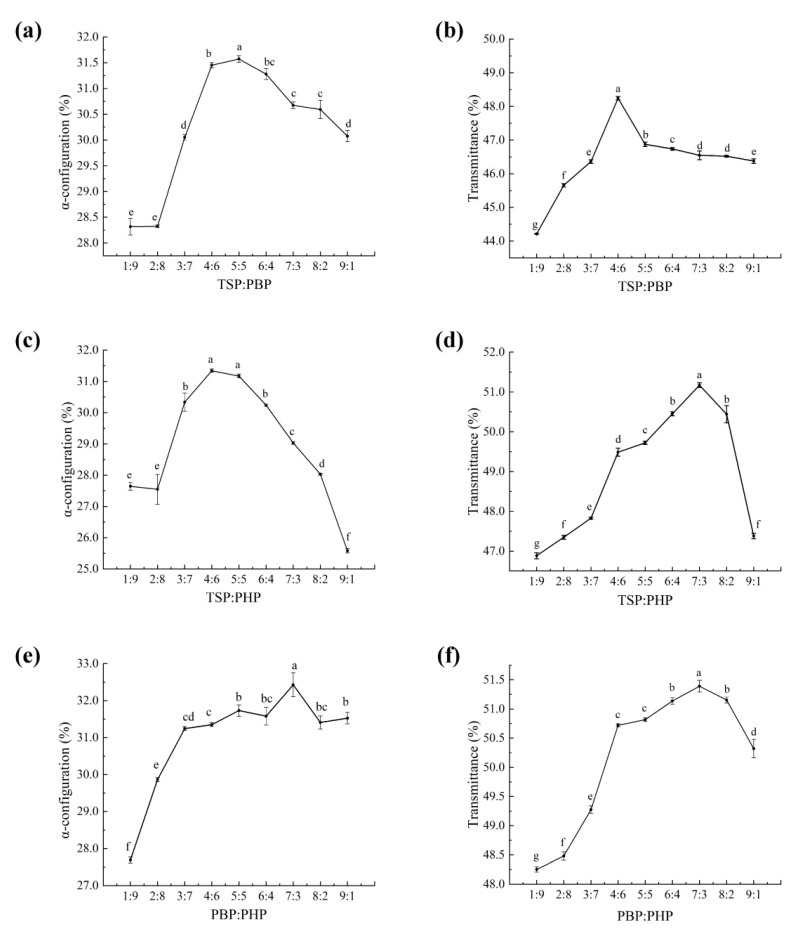
Effect of α-conformation and transmittance of hair in DH after treatment with different proportions (**a**–**f**) of TSP, PBP, and PHP. Lines (mean ± std dev, n = 3) with different letters indicate mean values that are significantly different (*p* < 0.05).

As shown in Figure 1a, the proportion of α-configuration (α-helix and irregular coil) in hair after DH treatment increases more rapidly when the TSP–PBP ratio is 3:7, reaches a peak of 31.62% at a 5:5 ratio, and then remains stable at 6:4, 7:3, and 8:2. Similarly, in Figure 1b, the α-configuration proportion also rises rapidly at a 3:7 ratio, peaks at 31.62% when the ratio is 5:5, and stabilizes thereafter around 46.5%. Overall, the transmittance at the S=O characteristic peak near 1042 cm^−1^ for TSP and PBP increases most rapidly when the ratio is 4:6, reaches a maximum of 48.23%, and then stabilizes around 46.5%. The tested TSP–PBP ratios include 3:7, 4:6, 5:5, 6:4, 7:3, 8:2, and 9:1.

From Figure 1c, the α-configuration proportion in hair after DH treatment rises more rapidly when the TSP–PHP ratio is 3:7, peaks at 31.32% at 4:6, and then decreases. In Figure 1d, the transmittance at the S=O peak (near 1042 cm^−1^) increases sharply when the TSP–PHP ratio is 4:6, reaches a maximum of 51.21% at 7:3, and then gradually declines at 8:2 and 9:1. The selected TSP–PHP ratios include 3:7, 4:6, 5:5, 6:4, 7:3, and 8:2.

As shown in Figure 1e, the increase in α-configuration proportion in DH-treated hair is more gradual when the PBP–PHP ratio is 3:7 and remains relatively stable overall, reaching a maximum at a 7:3 ratio. In Figure 1f, the transmittance at the S=O peak near 1042 cm^−1^ for DH-treated hair with PHP and PBP at a 3:7 ratio increases rapidly and then stabilizes, peaking at 51.37% when the ratio is 7:3. The examined PHP–PBP ratios include 3:7, 4:6, 5:5, 6:4, and 7:3.

Based on the one-way analysis of different ratios of TSP and PBP, TSP and PHP, and PHP and PBP on the α-configuration (α-helix and irregular curl) and the transmittance at the characteristic S=O peak near 1042 cm^−1^ in DH after treatment, response surface analyses were conducted to determine the optimal ratios of TSP, PBP, and PHP. The factors and levels for the response surface analysis, as presented in Table 1, were used to further explore the most effective combination of these three fish-derived hydrolyzed protein peptides.

### 3.3. Response Surface Analysis

The experimental design and data analysis results obtained using Design-Expert 11 software are presented in Table S1. After performing regression fitting analysis of Table S1, with the number of TSP copies (A), PBP copies (B), and PHP copies (C) as variables and α-conformation (Y1) and transmittance of S=O at 1042 cm^−1^ (Y2) as indicators, a multivariate quadratic regression model was derived as follows:

Y1 = 29.68 + 2.65A − 0.2125B + 0.4888C − 0.0275AB − 0.7450AC + 0.1575BC + 0.9760A^2^ − 0.4915B^2^ − 1.06C^2^ (1)

Y2 = 50.45 + 1.18A + 0.2525B + 0.0913C − 0.4200AB − 0.4075AC − 0.6800BC + 0.0857A^2^ + 0.1382B^2^ − 2.13C^2^ (2)

The *p*-value (*p* <0.0001) indicates that the model is statistically significant, while the non-significant misfit term (*p* > 0.05) suggests a good model fit with minimal deviation from the true value. Additionally, the regression equations were tested for significance, with the results presented in Tables S2 and S3.

**Figure 2:**
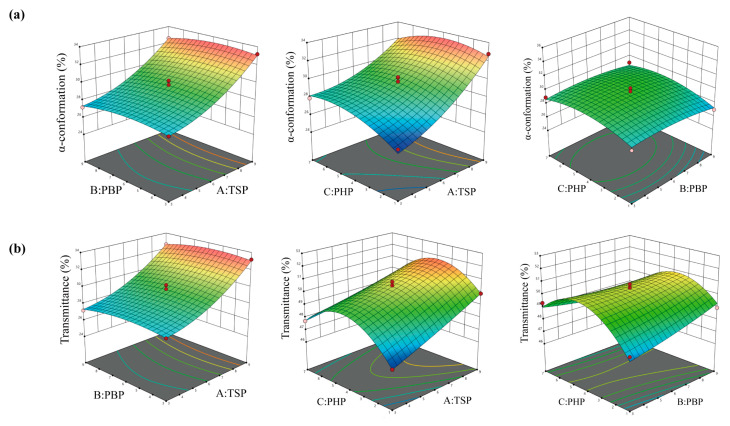
Effect of pair-to-pair interaction with DH on α-conformation (**a**) and transmittance (**b**) of hair in DH after treatment with different proportions of TSP, PBP, and PHP.

The response surface results are presented in Figure 2. The optimal conditions were verified using Design-Expert 11 software, which identified the ideal ratio for hair repair as TSP–PBP–PHP = 9:3.64:3.97. Under these conditions, the theoretical proportion of α-conformation (α-helix and irregular coil) of hair after DH treatment was 33.19%. Additionally, the theoretical transmittance of the S=O characteristic peak at 1042 cm^−1^ was 52.03%. This transmittance was superior to that observed in hair treated with other TSP and PHP ratios, TSP and PBP ratios, and PHP and PBP ratios. Moreover, it was closer to the values observed in NH.

### 3.4. Formulation of Fish-Derived Protein Peptide CHFREs

According to previous findings, the optimal ratio of the three fish-derived protein peptides was TSP–PBP–PHP = 9:3.64:3.97, which resulted in the most effective repair of DH. First, the CHFRE base was blended according to Table 6, followed by the addition of fish collagen peptide mixtures at different concentrations, as specified in Table 7.

The mixture of ingredients in Table 6 was heated in a water bath to 75 °C until fully melted, then homogenized using a homogenizer (IKA T10, IKA Company, Staufen, Germany) at 10,000 r/min for 25 min. Subsequently, it was stirred at the highest speed with a magnetic stirrer for another 25 min until a uniform mixture was achieved. The system was then cooled to 45 °C, at which point different concentrations of TSP–PBP–PHP were added one by one and stirred until homogeneous. Emulsion samples prepared with varying concentrations of TSP–PBP–PHP, as specified in Table 6 and Table 7, are shown in Figure S5.

According to rheological analysis, CHFRE exhibits a relatively high viscosity, facilitating uniform application (Figure S8a). Under sufficient applied stress, CHFRE transitions into a flowable state while maintaining stability, preventing excessive viscosity or undesirable loss (Figure S8b). The dominance of G′ over G″ indicates superior viscoelastic stability, effectively preventing phase separation (Figure S8c). These rheological properties suggest that the emulsion meets the requirements for a haircare formulation and is suitable for use as a hair conditioning emulsion. Infrared and rheological analyses were performed on the fabricated CHFRE. The infrared spectrum (Figure S9) shows that CHFRE contains protein or peptide structures (amide I: 1650 cm^−1^, amide II: 1540 cm^−1^) and strong hydrogen bonding (3400 cm^−1^), indicating the presence of a possible polymer network structure. The broad and intense peak in the 3500 cm^−1^–3000 cm^−1^ range suggests that the polymer material has long molecular chains, with enhanced hydrogen bonding and van der Waals forces both intramolecularly and intermolecularly, resulting in broader absorption peaks, which is characteristic of polymer emulsions.

### 3.5. Particle Size and Zeta Potential of Lyophilized Powders of TSP, PBP, PHP

As shown in Table 8, TSP has a small particle size, with the majority ranging between 100 and 500 nm, making it more easily penetrable into the hair cuticle and cortex. This facilitates the repair of internal hair structures and enhances the stability of the keratin network. Meanwhile, PBP and PHP exhibit significantly larger particle size distributions, particularly PBP, which contains a substantial proportion of particles exceeding 500 nm. These larger particles can adhere to the hair cuticle surface, forming a protective film that enhances shine and smoothness. Additionally, they contribute to the emulsification stability of the formulation, preventing phase separation and sedimentation [38,39].

As shown in Table 9, the zeta potential of TSP is negative (−23.556 mV), indicating that it carries a negative charge in solution. When in contact with hair, which typically has a certain positive charge on its surface, this negatively charged protein can bind to the hair through electrostatic attraction. The negative charge enhances the affinity between the protein and the hair, especially in improving the smoothness and strengthening its repairing effects, making it suitable for haircare products. The low conductivity (0.018 mS/cm) indicates low ionic strength in solution, minimizing interference with other ions or molecules and helping maintain a desirable adsorption state.

The zeta potential of PHP was −26.71 mV, which is more negative than that of the river herring skin protein hydrolysate, suggesting a stronger negative charge in the solution. This may result in greater electrostatic attraction when binding to the hair surface. The stronger negative charge could allow it to form a more stable adsorption layer on the hair, enhancing haircare benefits such as antistatic, moisturizing, and smoothing effects [40]. Its slightly higher conductivity (0.02954 mS/cm) indicates a slightly increased ionic strength, which may influence its stability under different conditions.

The zeta potential of PBP was −25.88 mV, with a negative charge and strong electrostatic interactions with the hair surface. Its negative charge was slightly higher than that of TSP but slightly lower than that of PHP, suggesting that it binds effectively to hair. The relatively high conductivity (0.06427 mS/cm) indicates strong ion mobility in the solution, which may somewhat affect the stability of the protein in the solution.

All three protein hydrolysates have the potential to enhance interactions with the hair surface, and the system’s conductivity remains relatively stable. Therefore, they can be incorporated into CHFREs to provide a repairing and smoothing effect on the hair [41].

### 3.6. Evaluation of Haircare Effect After Treatment of DH

#### 3.6.1. Analysis of Optical Microscopy Results

**Figure 3:**
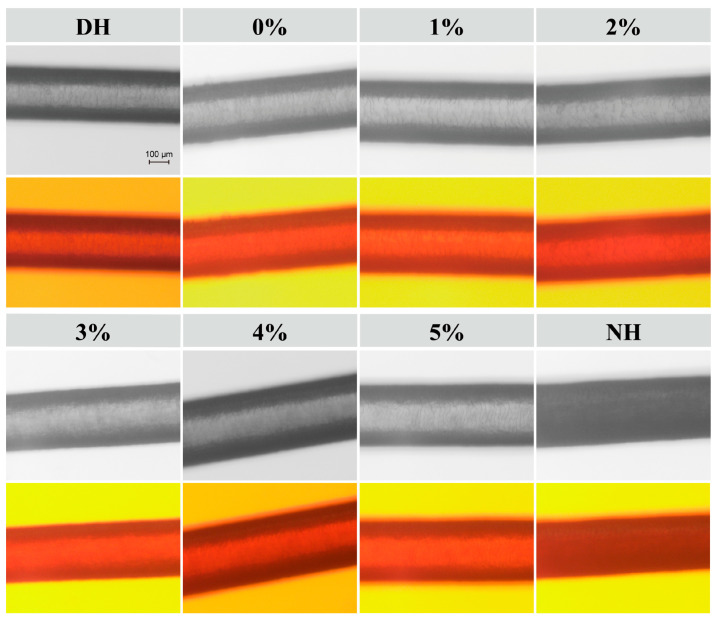
Optical microscopy of DH and NH after haircare treatment with different concentrations of TSP–PBP–PHP addition.

Based on the previously determined optimal ratio of TSP–PBP–PHP for DH repair, different concentration gradients were set to explore the optimal TSPP–PBP–PHP concentration. Figure 3 illustrates the surface morphology changes of DH and NH after conditioning treatment with varying TSPP–PBP–PHP additions.

Optical microscopy revealed that NH exhibited a glossier surface, intact scales, and a full, undamaged hair core, while DH lacked gloss, had disorganized scales, and displayed a vacant hair core. CHFRE without TSP–PBP–PHP showed no improvement in hair shine or core integrity. However, the addition of TSP–PBP–PHP to the CHFRE had different positive effects on the gloss and smoothness of the DH surface. The greater the amount added, the more pronounced the improvement, with the most significant enhancement observed at 5%, bringing the hair condition closer to that of normal hair.

#### 3.6.2. Analysis of SEM Results

**Figure 4:**
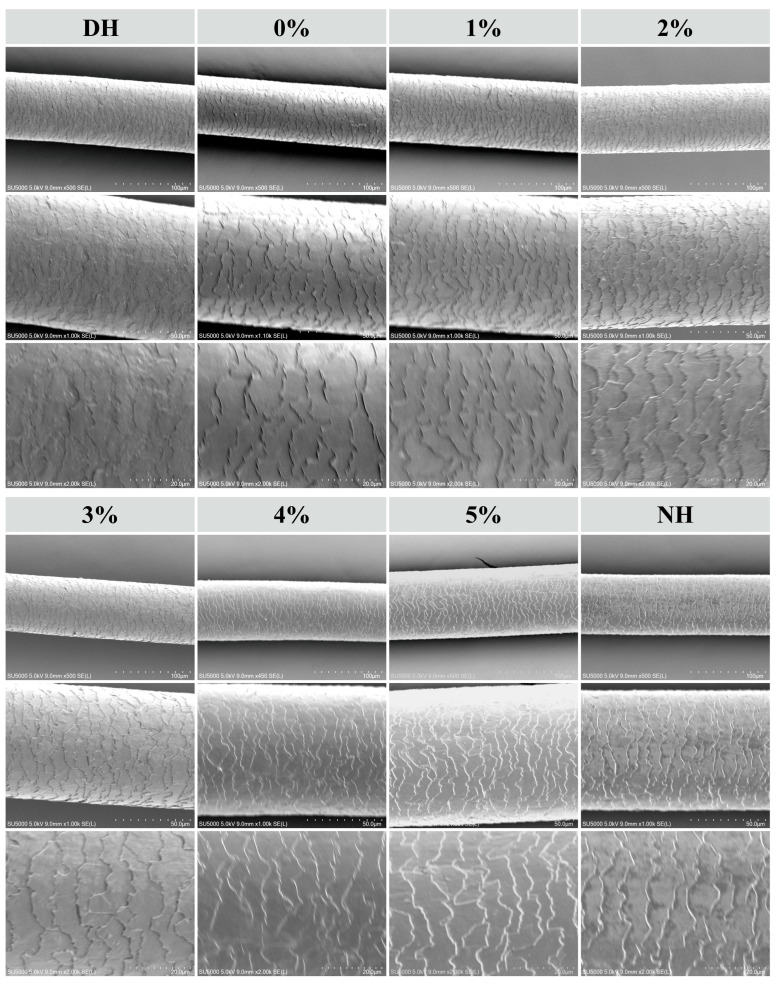
SEM images of DH and NH magnified by 500×, 1000×, and 2000× after haircare treatment with different concentrations of TSP–PBP–PHP addition.

The main chemical component of the hair cuticle is keratin, which forms a tile-like or scale-like structure. This protein structure is cross-linked by sulfation, creating a highly stable network that effectively protects hair strands from external damage, prevents water loss, and maintains the internal integrity of the hair [42]. Figure 4 presents scanning electron microscope images of DH and NH hair at magnifications of 500×, 1000×, and 2000× after CHFRE treatment with varying TSP–PBP–PHP concentrations. The magnified images of the hair cuticle morphology clearly illustrate a gradual transition in the emulsion’s repair effect from DH to NH as the concentration increases.

It was observed that the cuticle surface scales of NH were neatly arranged, highly smooth, and free of debris. No warping or flipping was detected, and the hair surface exhibited a noticeable luster. In contrast, the cuticle surface scales of DH were disrupted and disordered, with an unclear texture, numerous voids and cracks, and a dark, dull appearance. CHFRE treatment with 1–5% TSP–PBP–PHP improved DH cuticle structure, with higher concentrations yielding greater improvements. Increased application resulted in better alignment and restoration of hair scales. CHFREs without TSP–PBP–PHP, as well as those containing 1%, 2%, and 3% TSP–PBP–PHP, showed improvements in the cuticle scales, though to a lesser extent than those with 4% and 5% TSP–PBP–PHP. Moreover, the 5% TSP–PBP–PHP formulation produced cuticle scales most similar to those of normal hair, effectively closing previously curled and flared scales and arranging them in a neater, more regular pattern. Therefore, it can be concluded that the application of CHFRE with TSP–PBP–PHP contributes to repairing surface damage and aligning the cuticle scales. Further analysis of internal chemical composition changes is necessary to better understand this effect. Compared to rinse-off conditioners containing argan oil or camellia oil [42], CHFRE exhibits a more rapid repair effect on damaged hair while promoting a more orderly and smoother arrangement of the hair cuticle. Compared to modified wheat gluten protein shampoo [43], which exhibits a certain degree of efficacy in repairing hair cuticles, its performance remains slightly inferior to that of CHFRE containing a high concentration of TSP–PBP–PHP. Following CHFRE treatment, the hair cuticles appear smoother, more compact, and more uniformly arranged, with a clearer structural definition, demonstrating superior repair efficacy.

#### 3.6.3. Analysis of Differential Calorimetric Scanning Results

Table 10 illustrates the effect of different concentrations of TSP–PBP–PHP added to t CHFRE on the α-helix peak of hair. The denaturation of the α-helical structure is typically accompanied by a heat-absorbing transformation process, which appears as an endothermic peak in the DSC curves, referred to as the denaturation peak (Td, °C) in Table 10 [44]. The denaturation enthalpy (ΔH_d_) at this peak represents the amount of heat required to transition the protein from the folded, stable α-helix conformation to an unfolded state. The RHC indicates a comparative value with DH. If RHC > 1, the treated sample absorbs more heat than the untreated sample, implying greater structural stability. Conversely, if RHC < 1, the treated sample absorbs less heat. The results indicate that as the concentration increases, the RHC value also rises. The data suggest that the 5% concentration is optimal for repairing damaged hair, making it closest to normal hair. Combined with the analysis of protein secondary structure and water retention, an increase in protein peptide concentration facilitates deeper protein penetration into the hair fiber, leading to enhanced repair of damaged α-keratin conformation and improved fiber structural stability [45].

#### 3.6.4. Analysis of Infrared Spectral Results

**Figure 5:**
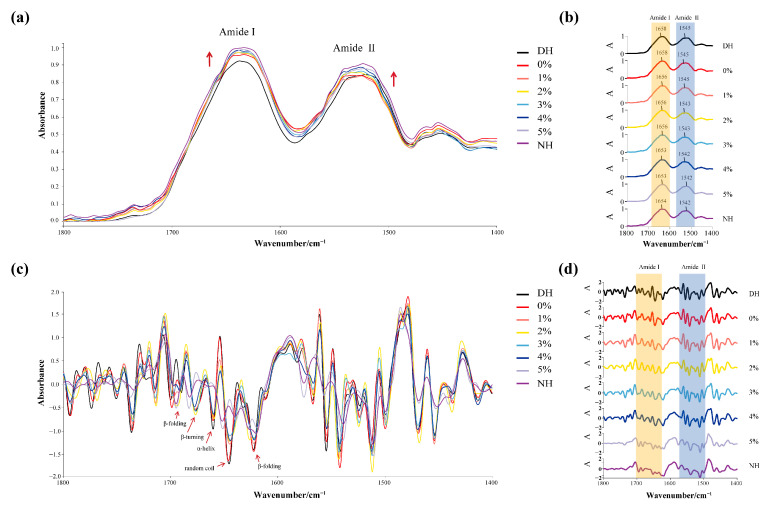
IR spectra of DH care with different concentrations of TSP–PBP–PHP addition. (**a**) The infrared average spectral fold of the hair in the protein band; (**b**) the resolution of the infrared average spectrum of the hair in the protein band; (**c**) infrared second-derivative spectra of hair in the protein band; (**d**) split plot of infrared second-derivative spectrogram of hair in protein band.

Hair possesses both a microstructure and a macrostructure, with various chemical bonds, including peptide bonds (-CO-NH-), disulfide bonds (-S-S-), hydrogen bonds, salt bonds, hydrophobic forces, and other forces [46]. The wave number segments corresponding to the amide I (1700–1600 cm^−1^) and amide II (1600–1500 cm^−1^) bands in unprocessed damaged hair are significantly higher than those in normal hair [47], which aligns with previous findings from our research group [48]. The overlaid infrared average spectrograms (Figure 5a) of DH treated with different concentrations of TSP–PBP–PHP in the protein band (1800–1400 cm^−1^), along with their respective spectrogram splits (Figure 5b), indicate that compared to untreated DH, the peaks of the amide I band at 1662–1658 cm^−1^ and 1650–1648 cm^−1^ increased significantly as the protein concentration rose. The segment peaks of the amide I band at 1648 cm^−1^ and the amide II bands at 1650^−1^ also showed a marked increase, gradually approaching NH. This prevented smaller IR absorption peaks from being masked, while second-order derivative processing amplified the signals to highlight subtle protein changes. As shown in Figure 5c,d, the peak shape of DH in the amide I band (1700–1600 cm^−1^) became flatter after treatment with different concentrations of TSP–PBP–PHP, and the number of peaks decreased compared to untreated DH, making it more similar to NH.

The α-helix [32,33] and β-folding keratin conformation are crucial parameters for evaluating the effectiveness of haircare products. Keratin conformation serves as a direct molecular indicator of hair’s appearance and internal structure. Active ingredients capable of modulating keratin conformation by converting β-folding [34] conformations into α-helix conformations are considered effective hair-smoothing agents at the molecular level. The amide I bands were analyzed by deconvolution and second-order derivative fitting to quantify the changes in the relative content of the secondary structure of DH after CHFRE treatment. As shown in Table 11, untreated DH exhibited severe damage, with a 6.52% decrease in the α-configuration (α-helix and random coil). Ignoring sample differences, the majority of the lost α-configuration was converted into β-configuration (β-turning and β-antiparallel), increasing by 6.85%. As the concentration of TSP–PBP–PHP declined, the proportion of α-conformations rose while the β-conformations decreased, with the 5% concentration yielding the best results. This optimal performance at 5% TSP–PBP–PHP is further corroborated by the gradual red shift observed in the amide I and II bands in Figure 5 of the one-dimensional infrared spectra.

**Figure 6:**
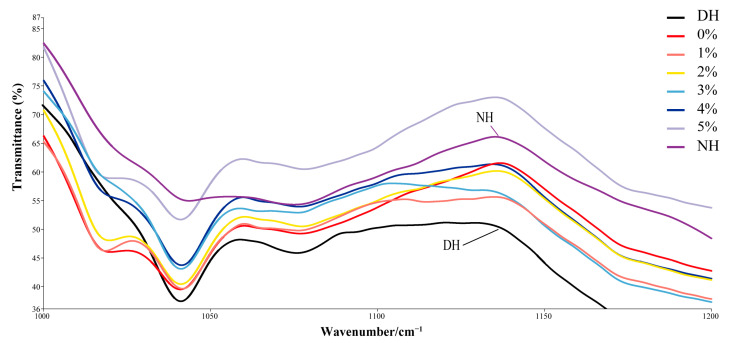
Infrared mean spectra of cystine derivatives in DH after treatment with different concentrations of TSP–PBP–PHP addition.

**Figure 7:**
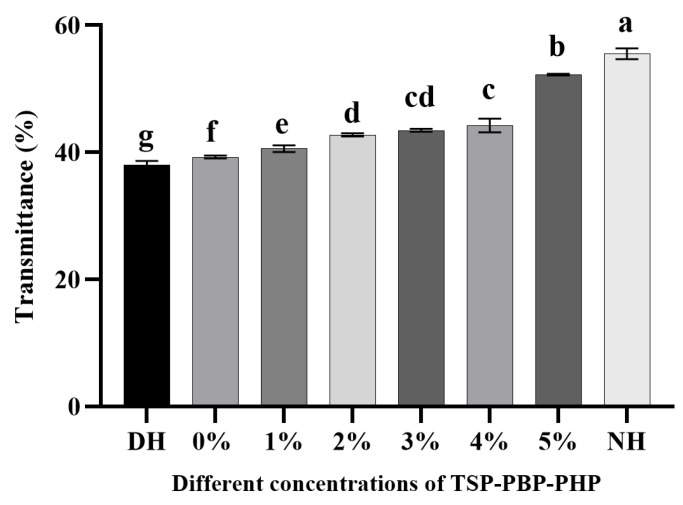
The transmittance of S=O at 1042 cm^−1^ after DH treatment by different concentrations of TSP–PBP–PHP addition. Bars (mean ± std dev, n = 3) with different letters indicate mean values that are significantly different (*p* < 0.05).

The characteristic peaks of S=O, observed near 1042 cm^−1^, correspond to sulfenic acid and sulfonic acid groups, which result from the oxidative degradation of disulfide bonds [35]. In short, the sharper and more pronounced these characteristic peaks, the more severe the oxidative damage. As shown in Figure 6, normal hair exhibits a nearly invisible and smooth S=O characteristic peak, whereas DH displays a strong and distinct signal, indicating significant oxidative damage. The application of CHFRE with varying concentrations of TSP–PBP–PHP progressively weakens this S=O characteristic peak. As shown in Figure 7, the quantitative analysis of S=O transmittance at 1042 cm^−1^ further confirms that transmittance increases with increasing concentration. The transmittance of damaged hair was significantly lower than that of all treatment groups. Although the group without TSP–PBP–PHP showed some improvement, the increase was not statistically significant. With increasing TSP–PBP–PHP concentrations, transmittance exhibited a significant upward trend, with the 5% TSP–PBP–PHP group showing significantly higher transmittance than the 1–4% groups, indicating the most effective repair performance. These findings suggest that CHFRE has the potential to mitigate oxidative damage in hair.

#### 3.6.5. Analysis of Hair Amino Acid Results

**Figure 8:**
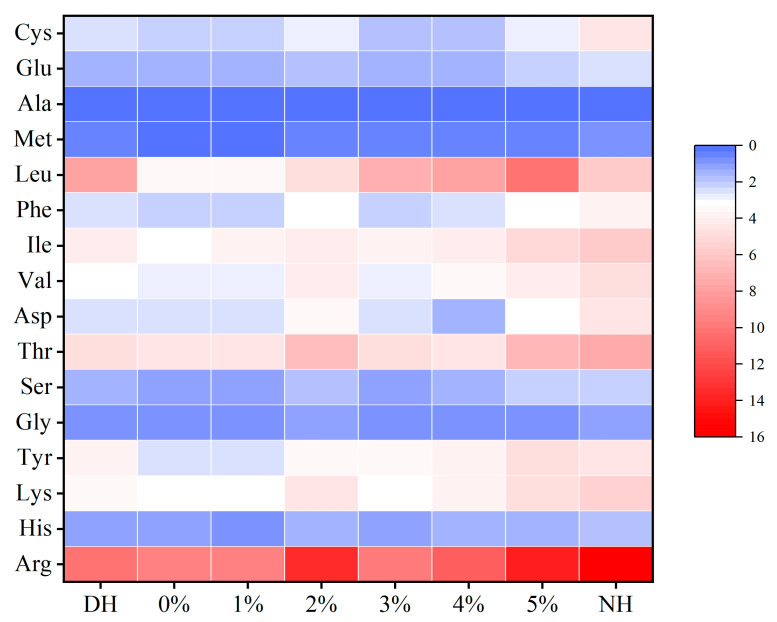
Changes in amino acid content after DH treatment by haircare with different concentrations of TSP–PBP–PHP addition (g/100 g).

Hair is primarily composed of protein, so changes in the amino acid composition can significantly affect the structure and properties of hair keratin [49]. Table 12 presents the changes in amino acid classes and contents of CHFREs at different concentrations of TSP–PBP–PHP after DH treatment, while Figure 8 provides a heat map illustrating these changes. It can be observed that both individual amino acid levels and the total amino acid content of hair changed after DH treatment with various concentrations of TSP–PBP–PHP.

Cystine forms disulfide bonds that enhance the mechanical strength and toughness of hair [50]. NH has a higher Cys content, whereas DH shows a reduced Cys level, indicating structural damage from bleaching and dyeing. Treatment with 5% TSP–PBP–PHP results in the highest Cys content, suggesting that higher concentrations of mixed protein peptides promote disulfide bond formation, thereby improving hair toughness. This observation aligns with previous findings on disulfide bond transmittance. It has been reported that Glu, Met, Ala, and Leu contribute to the formation and stabilization of the α-helical structure in hair strands [49]. As shown in Figure 8, their content increases with higher mixed proteo-peptide concentrations, approaching the levels observed in NH. The 5% TSP–PBP–PHP treatment yields amino acid levels closest to those in NH. Hydrophobic amino acids (Ala, Val, Met, Ile, Leu, and Phe) enhance the interaction with hair and are crucial in maintaining the α-helical keratin structure in intermediate filaments [51]. Their content in DH is significantly lower than in NH and increases with higher concentrations of TSP–PBP–PHP, reaching levels comparable to NH at 5% concentration. A similar trend is observed for total amino acid content.

## 4. Conclusions

Existing commercial haircare products generally exhibit limited repair efficacy (Figure S10 and Table S5). While some products partially seal hair cuticles and smooth the hair, in some cases, they exacerbate hair roughness due to the excessive surfactant content. Additionally, although silicone-based formulations may enhance hair texture, they hinder the absorption of protein peptides and amino acids, thereby compromising internal structural repair. Most products fail to effectively stabilize the α-configuration or facilitate keratin reorganization, resulting in overall suboptimal restorative performance. Even when certain products increase amino acid content, their penetration and binding capacities remain insufficient, limiting their ability to provide deep repair and long-lasting nourishment.

In comparison, the emulsion treatment described in this study exhibited exceptional restorative effects on DH, effectively reducing breakage, sealing hair cuticles, and filling pores caused by oxidative damage. The peptide/protein analogs in the emulsion were able to penetrate the cortical layer to varying degrees, enhancing protein absorption and optimizing its distribution, while the cystine derivatives progressively exhibited absorption characteristics similar to those of healthy hair. Following CHFRE treatment, the α-configuration content increased by 5.94%, and the RHC increased by 28.44%, significantly enhancing protein stability and bringing the keratin structure closer to that of healthy hair. Additionally, the transmittance of the S=O characteristic peak at 1042 cm^−1^ increased by 14.63%, further confirming a reduction in oxidative damage. Furthermore, total amino acid content increased by 16.77 g/100 g and hydrophobic amino acid content increased by 2.47 g/100 g, indicating improved hair affinity and keratin stability. Overall, CHFRE substantially improved the structural and chemical stability of damaged hair, enhanced protein absorption and uniform distribution, effectively mitigated oxidative damage, and increased amino acid content, making the hair more comparable to its healthy counterpart. This study provides valuable insights for the development of natural hair repair products with enhanced efficacy.

This study provides a novel approach to the efficient utilization of natural polymer proteins in the development of sustainable and eco-friendly haircare materials, contributing to the advancement of green beauty innovations and the sustainable use of resources.

## Figures and Tables

**Table 1:** Factors and levels of TSP, PBP, and PHP intestines in the response surface experiment.

Level	Factors		
	TSP Number	PBP Number	PHP Number
−1	3	3	1
0	6	6	4
1	9	9	7

**Table 2:** Percentage of protein secondary structures in DH after treatment with different proportions of TSP and PBP.

Secondary Structure (%)	β-Folding	Random Coil	α-Helix	β-Turning	β-Antiparallel	α-Conformation	β-Conformation
DH	40.16	13.99	13.40	27.28	5.17	27.39	72.61
TSP:PBP = 1:9	38.71	14.47	13.78	27.95	5.08	28.25	71.74
TSP:PBP = 2:8	38.52	14.53	13.82	27.99	5.13	28.35	71.64
TSP:PBP = 3:7	37.27	15.47	14.58	23.61	9.07	30.05	69.95
TSP:PBP = 4:6	44.04	16.15	15.31	18.76	5.75	31.46	68.55
TSP:PBP = 5:5	43.61	16.11	15.51	18.60	6.17	31.62	68.38
TSP:PBP = 6:4	44.43	15.95	15.29	18.52	5.80	31.24	68.75
TSP:PBP = 7:3	45.06	15.88	14.84	18.26	5.96	30.72	69.28
TSP:PBP = 8:2	44.96	15.77	14.71	18.94	5.62	30.48	69.52
TSP:PBP = 9:1	45.59	15.75	14.27	18.91	5.48	30.02	69.98
NH	31.29	17.38	16.53	27.09	7.71	33.91	65.76

**Table 3:** Percentage of protein secondary structures in DH after treatment with different proportions of TSP and PHP.

Secondary Structure (%)	β-Folding	Random Coil	α-Helix	β-Turning	β-Antiparallel	α-Conformation	β-Conformation
DH	40.16	13.99	13.40	27.28	5.17	27.39	72.61
TSP:PHP = 1:9	39.15	14.07	13.66	27.95	5.16	27.73	72.26
TSP:PHP = 2:8	39.51	13.97	13.87	27.37	5.28	27.84	72.16
TSP:PHP = 3:7	39.44	15.39	15.12	22.84	7.22	30.51	69.50
TSP:PHP = 4:6	44.32	15.60	15.72	18.20	6.15	31.32	68.67
TSP:PHP = 5:5	44.41	15.33	15.88	17.87	6.51	31.21	68.79
TSP:PHP = 6:4	33.83	15.43	14.84	30.60	5.30	30.27	69.73
TSP:PHP = 7:3	37.18	15.11	13.97	29.13	4.61	29.08	70.92
TSP:PHP = 8:2	38.76	14.39	13.67	28.38	4.81	28.06	71.95
TSP:PHP = 9:1	41.88	13.42	12.22	21.67	10.81	25.64	74.36
NH	31.29	17.38	16.53	27.09	7.71	33.91	65.76

**Table 4:** Percentage of protein secondary structures in DH after treatment with different proportions of PBP and PHP.

Secondary Structure (%)	β-Folding	Random Coil	α-Helix	β-Turning	β-Antiparallel	α-Conformation	β-Conformation
DH	40.16	13.99	13.40	27.28	5.17	27.39	72.61
PBP:PHP = 1:9	39.19	14.05	13.71	27.78	5.27	27.76	72.24
PBP:PHP = 2:8	42.27	14.78	15.1	18.43	6.43	29.88	67.13
PBP:PHP = 3:7	44.32	15.60	15.63	18.19	6.26	31.23	68.77
PBP:PHP = 4:6	41.97	15.99	15.35	17.97	8.71	31.34	68.65
PBP:PHP = 5:5	31.18	14.93	16.76	25.83	11.41	31.69	68.42
PBP:PHP = 6:4	44.22	15.90	15.83	18.07	5.97	31.73	68.26
PBP:PHP = 7:3	43.32	15.99	16.29	18.22	6.19	32.28	67.73
PBP:PHP = 8:2	44.19	15.78	15.74	18.04	6.24	31.52	68.47
PBP:PHP = 9:1	44.41	15.74	15.73	17.98	6.14	31.47	68.53
NH	31.29	17.38	16.53	27.09	7.71	33.91	65.76

**Table 5:** Transmittance of S=O at 1042 cm^−1^ after Treatment of DH with Different Proportions of TSP, PBP, and PHP.

Transmittance (%)	TSP: PBP	TSP:PHP	PBP:PHP
1:9	44.22	46.90	48.27
2:8	45.67	47.34	48.54
3:7	46.39	47.80	49.32
4:6	48.23	49.46	50.70
5:5	46.92	49.77	50.85
6:4	46.73	50.45	51.11
7:3	46.64	51.21	51.37
8:2	46.50	50.62	51.16
9:1	46.43	47.43	50.26

**Table 6:** Base formula sheet of CHFRE (Unit: g).

Element	Purpose	Quantity
deionized water	solvent	88.1–83.9
Ceteartrimonium Chloride	Cationic surfactants; Antistatic; Antimicrobial agents	7.0–8.0
CETEARYL ALCOHOL	Emulsifier; Softener; Thickener	3.0–4.0
EDTA-2Na [36,37]	Chelating agent	0.1–0.2
CL12490	Coloring agent	0.2–0.3
CL11680	Coloring agent	0.2–0.3
Citric Acid (adjust of pH ≈ 5)		0.3–0.4

**Table 7:** TSP–PBP–PHP additive concentration in CHFRE (Unit: g).

TSP-PBP-PHP Additive Concentration	0%	1%	2%	3%	4%	5%
CHFRE base additions	50.0	49.5	49.0	48.5	48.0	47.5
TSP-PBP-PHP additions	0.00	0.50	1.00	1.50	2.00	2.50
TSP additions	0.000	0.271	0.542	0.813	1.084	1.354
PBP additions	0.000	0.109	0.219	0.328	0.438	0.478
PHP additions	0.000	0.119	0.239	0.358	0.548	0.597

**Table 8:** The particle size distribution of TSP, PBP, and PHP.

Size Proteins Percentage (%)	TSP	PBP	PHP
Small size (100–200 nm)	36.35	-	-
Medium size (200–500 nm)	63.62	-	1.01
Large size (500–1000 nm)	1.35	61.20	30.68
Extremely large size (>1000 nm)	-	38.81	68.31

**Table 9:** Zeta potential of TSP, PBP, and PHP.

	Zeta Potential (mV)	Conductivity (mS/cm)	Quality Factor
TSP	−23.56	0.018	1.66
PHP	−26.71	0.030	1.82
PBP	−25.88	0.064	1.55

**Table 10:** Effect of haircare with different concentrations of TSP–PBP–PHP addition on hair α-spiral peak after DH treatment.

	DH	0%	1%	2%	3%	4%	5%	NH
T_d_ (°C)	243.92	242.03	244.21	238.63	243.20	244.09	245.83	249.87
ΔH_d_ (J/g)	5.707	5.466	6.115	6.518	6.721	7.127	7.330	10.460
RHC	100.00%	95.78%	107.15%	114.21%	117.77%	124.80%	128.44%	183.28%

**Table 11:** Percentage of protein secondary structures in DH after treatment with different concentrations of TSP–PBP–PHP addition.

Secondary Structure (%)	DH	0%	1%	2%	3%	4%	5%	NH
β-folding	40.16	40.99	47.72	45.78	41.53	44.68	39.00	31.29
random coil	13.99	15.57	15.44	16.27	16.45	16.43	17.27	17.38
α-helix	13.40	12.94	13.55	14.26	14.62	15.18	16.06	16.53
β-turning	27.28	25.92	18.59	19.17	18.98	18.37	21.85	27.09
β-antiparallel	5.17	3.56	4.69	4.51	8.12	5.64	5.81	7.71
α-conformation	27.39	28.51	28.99	30.53	31.07	31.61	33.33	33.91
β-conformation	72.61	70.47	71.00	69.46	68.63	68.69	66.66	65.76

**Table 12:** Changes in total amino acids and hydrophobic amino acids after DH treatment by haircare with different concentrations of TSP–PBP–PHP addition (g/100 g).

Amino Acid Types	DH	0%	1%	2%	3%	4%	5%	NH
Cys	2.38 ± 0.02	2.16 ± 0.06	2.16 ± 0.06	2.97 ± 0.09	1.99 ± 0.09	1.87 ± 0.03	2.96 ± 0.06	4.45 ± 0.08
Glu	1.54 ± 0.07	1.47 ± 0.01	1.47 ± 0.04	1.99 ± 0.08	1.48 ± 0.01	1.58 ± 0.01	2.21 ± 0.06	2.37 ± 0.08
Ala *	0.14 ± 0.06	0.19 ± 0.01	0.21 ± 0.05	0.25 ± 0.04	0.08 ± 0.09	0.24 ± 0.02	0.23 ± 0.08	0.23 ± 0.06
Met *	0.40 ± 0.05	0.33 ± 0.09	0.33 ± 0.09	0.53 ± 0.05	0.37 ± 0.06	0.46 ± 0.05	0.50 ± 0.02	0.70 ± 0.01
Leu *	7.92 ± 0.36	3.51 ± 0.08	3.51 ± 0.08	4.93 ± 0.07	7.30 ± 0.06	7.94 ± 0.05	10.07 ± 0.04	5.76 ± 0.04
Phe *	2.54 ± 0.06	2.25 ± 0.03	2.25 ± 0.03	3.21 ± 0.07	2.29 ± 0.02	2.53 ± 0.02	3.27 ± 0.08	3.80 ± 0.02
Ile *	4.06 ± 0.03	3.13 ± 0.06	3.69 ± 0.01	4.10 ± 0.05	3.85 ± 0.07	4.22 ± 0.02	5.31 ± 0.01	5.98 ± 0.05
Val	3.17 ± 0.02	2.89 ± 0.02	2.89 ± 0.02	4.16 ± 0.07	2.92 ± 0.07	3.54 ± 0.01	4.23 ± 0.08	4.67 ± 0.03
Asp	2.38 ± 0.03	2.5 ± 0.03	2.55 ± 0.03	3.54 ± 0.09	2.54 ± 0.07	1.60 ± 0.01	3.10 ± 0.07	4.36 ± 0.09
Thr	4.79 ± 0.06	4.6 ± 0.03	4.65 ± 0.06	6.54 ± 0.04	4.80 ± 0.02	4.61 ± 0.03	6.68 ± 0.06	7.45 ± 0.05
Ser	1.39 ± 0.09	1.30 ± 0.19	1.32 ± 0.04	1.69 ± 0.06	1.21 ± 0.07	1.36 ± 0.08	2.03 ± 0.06	2.19 ± 0.05
Gly	0.73 ± 0.02	0.70 ± 0.14	0.70 ± 0.04	1.00 ± 0.06	0.71 ± 0.09	0.67 ± 0.03	0.70 ± 0.02	1.14 ± 0.02
Tyr	3.77 ± 0.03	2.51 ± 0.01	2.51 ± 0.01	3.64 ± 0.07	3.63 ± 0.01	3.92 ± 0.06	4.90 ± 0.06	4.58 ± 0.01
Lys	3.36 ± 0.09	3.096 ± 0.03	3.09 ± 0.03	4.48 ± 0.03	3.22 ± 0.07	3.80 ± 0.08	4.68 ± 0.07	5.36 ± 0.08
His	1.19 ± 0.06	1.25 ± 0.05	0.99 ± 0.03	1.57 ± 0.04	1.08 ± 0.01	1.40 ± 0.01	1.64 ± 0.02	1.85 ± 0.09
Arg	10.22 ± 0.28	9.40 ± 0.06	9.40 ± 0.06	13.64 ± 0.03	9.94 ± 0.04	11.32 ± 0.03	14.27 ± 0.35	15.93 ± 0.07
Total amino acids	50.07	41.47	41.78	58.36	47.46	51.14	66.84	70.88
Hydrophobic amino acid	18.26	12.31	12.89	17.20	16.83	18.95	23.63	21.16

Note: * are hydrophobic amino acids.

## Data Availability

The original contributions presented in the study are included in the article and Supplementary Material; further inquiries can be directed to the corresponding author.

## Supplementary Materials

The following supporting information can be downloaded at: https://www.mdpi.com/article/10.3390/polym17070907/s1, Figure S1. IR-spectra of DH care with different proportions of TSP and PBP. (a) The infrared average spectral fold of the hair in the protein band. (b) Split plot of infrared second-derivative spectrogram of hair in protein band. Figure S2. IR spectra of DH care with different proportions of TSP and PHP. (a) On the left is the infrared average spectral fold of the hair in the protein band. (b) Split plot of infrared second-derivative spectrogram of hair in protein band. Figure S3. IR spectra of DH care with different proportions of PBP and PHP. (a) The infrared average spectral fold of the hair in the protein band. (b) Split plot of infrared second-derivative spectrogram of hair in protein band. Figure S4. (a) Infrared mean spectra of cystine derivatives in DH after treatment with different proportions of TSP and PBP. (b) Infrared mean spectra of cystine derivatives in DH after treatment with different proportions of TSP and PHP. (c) Infrared mean spectra of cystine derivatives in DH after treatment with different proportions of PBP and PHP. Figure S5. Emulsion samples made by adding different concentrations of TSP–PBP–PHP. Figure S6. DH and NH samples. Figure S7. Infrared spectra of TSP, PBP, and PHP. Figure S8. Rheological analysis of materials in CHFRE. (a) The effect of shear rate on the viscosity of CHFRE; (b) the effect of shear rate on the stress of CHFRE; (c) the effect of frequency on the storage modulus (G′) and loss modulus (G″) of CHFRE. Figure S9. Infrared spectra of CHFRE (5% TSP–PBP–PHP). Figure S10. SEM images of DH and NH magnified by 500×after haircare treatment with CHFRE (5% TSP–PBP–PHP) and commercial product (A, B, C, D, and E). Table S1. Box–Behnken design results of α-conformation and transmittance. Table S2. Analysis of variance and coefficient significance test of α-conformation regression model. Table S3. Analysis of variance and coefficient significance test of transmittance regression model. Table S4. Amino acid content of TSP, PBP, and PHP (g/100 g). Table S5. Comparison of repair indices between CHFRE and commercial product (A, B, C, D, and E).
