# Supplementary material for: Composite Fish Collagen Peptide-Based Biopolymer Emulsion for Keratin Structure Stabilization and Hair Fiber Repair
Source: Polymers (Basel). 2025 Mar 27;17(7):907. doi: 10.3390/polym17070907 (PMC11991457; doi:10.3390/polym17070907)
Supplement: Supplementary file 1 [file polymers-17-00907-s001.zip › polymers-3539792-supplementary.pdf]

polymers

MDPI

Supplementary Materials

Composite Fish Collagen Peptide-Based Biopolymer Emulsion for Keratin Structure Stabilization and Hair Fiber Repair

Wenwei Gu [1,†], Lei Gu [1,†], Ningping Tao [1,2,3,4], Xichang Wang [1,2,3,4] and Changhua Xu [1,2,3,4,*]

1 College of Food Science & Technology, Shanghai Ocean University, Shanghai 201306, China; m220300894@st.shou.edu.cn (W.G.); leigu1023@163.com (L.G.); nptao@shou.edu.cn (N.T.); xcwang@shou.edu.cn (X.W.)
2 Shanghai Engineering Research Center of Aquatic-Product Processing & Preservation, Shanghai 201306, China
3 Laboratory of Quality and Safety Risk Assessment for Aquatic Products on Storage and Preservation (Shanghai), Ministry of Agriculture, Shanghai 201306, China
4 National R&D Branch Center for Freshwater Aquatic Products Processing Technology, Shanghai 201306, China
* Correspondence: chxu@shou.edu.cn
† These authors contributed equally to this work and shared first author.

Academic Editor: Eduardo Guzmán

Received: 5 March 2025
Revised: 22 March 2025
Accepted: 26 March 2025
Published: 27 March 2025

Citation: Gu, W.; Gu, L.; Tao, N.; Wang, X.; Xu, C. Composite Fish Collagen Peptide-Based Biopolymer Emulsion for Keratin Structure Stabilization and Hair Fiber Repair. *Polymers* **2025**, *17*, 907. https://doi.org/10.3390/polym17070907

Copyright: © 2025 by the authors. Licensee MDPI, Basel, Switzerland. This article is an open access article distributed under the terms and conditions of the Creative Commons Attribution (CC BY) license (https://creativecommons.org/licenses/by/4.0/).

Figure

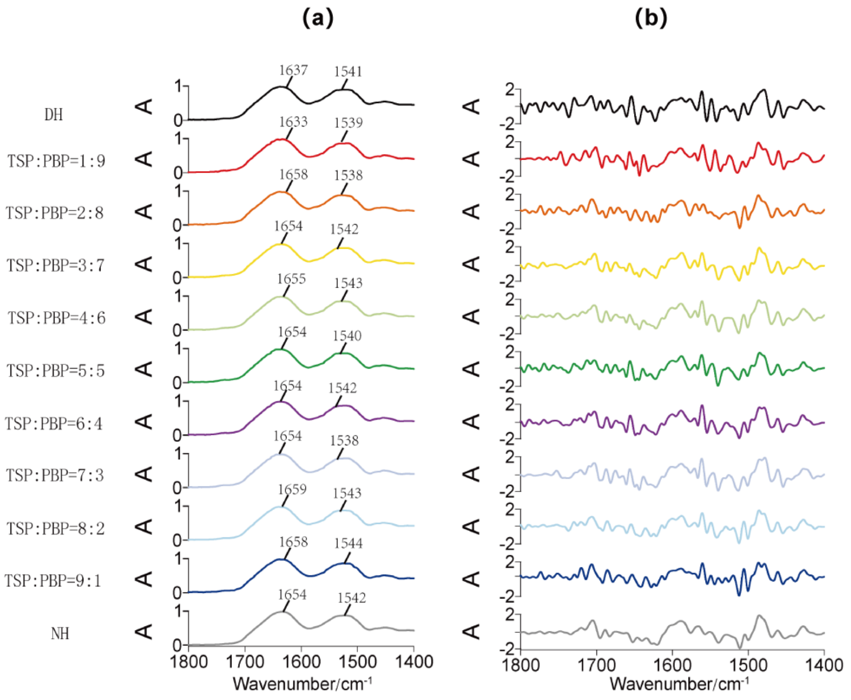

Figure S1. IR-spectra of DH care with different proportions of TSP and PBP. (a) The infrared average spectral fold of the hair in the protein band. (b) Split-plot of infrared second-derivative spectrogram of hair in protein band.

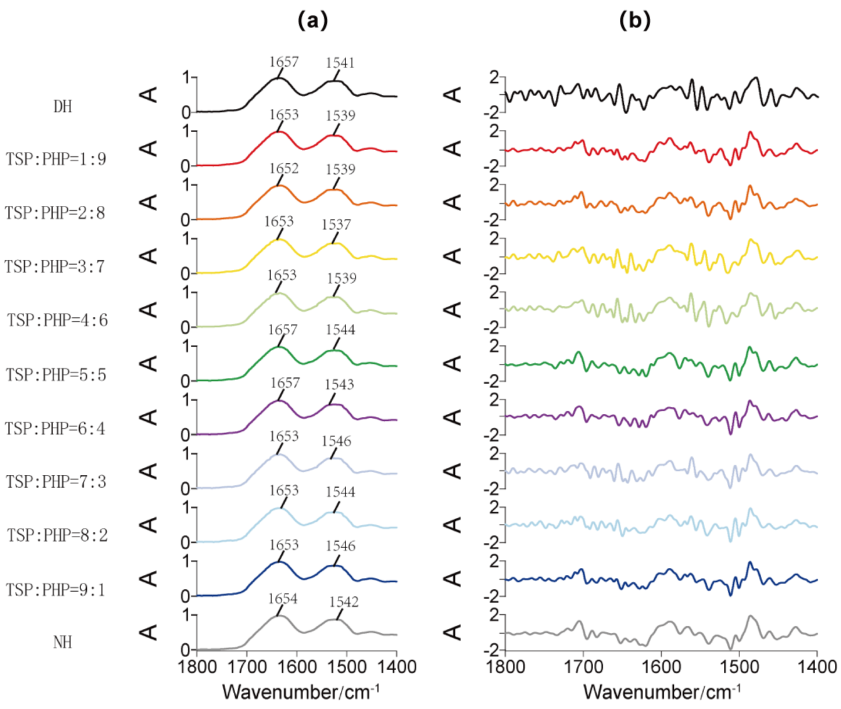

Figure S2. IR-spectra of DH care with different proportions of TSP and PHP. (a) On the left is the infrared average spectral fold of the hair in the protein band. (b) Split-plot of infrared second-derivative spectrogram of hair in protein band.

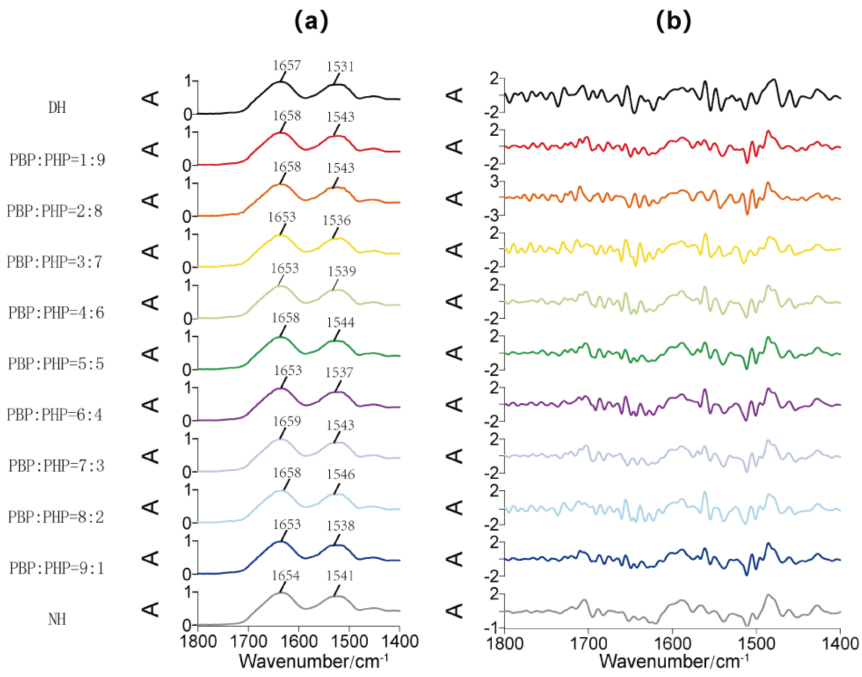

Figure S3. IR-spectra of DH care with different proportions of PBP and PHP. (a) The infrared average spectral fold of the hair in the protein band. (b) Split-plot of infrared second-derivative spectrogram of hair in protein band.

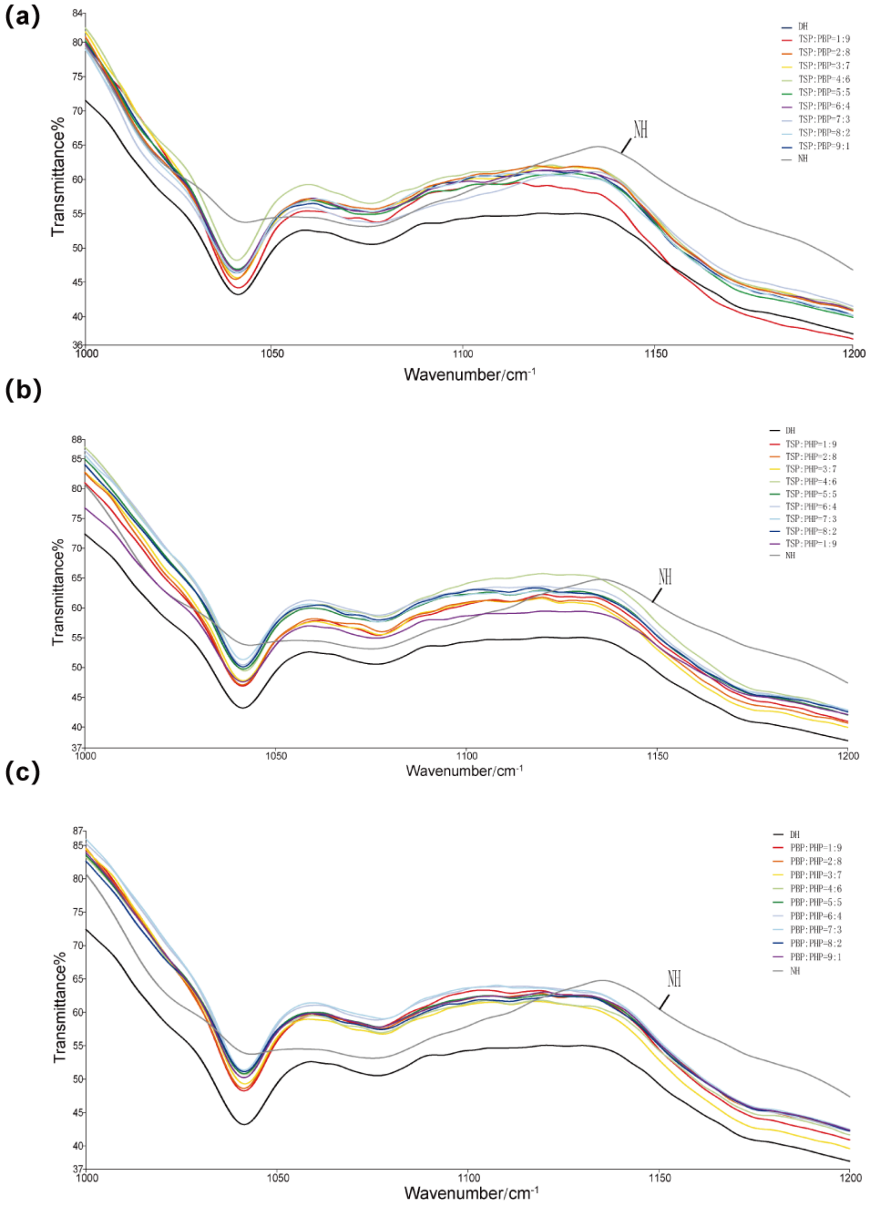

Figure S4. (a) Infrared mean spectra of cystine derivatives in DH after treatment with different proportions of TSP and PBP. (b) Infrared mean spectra of cystine derivatives in DH after treatment with different proportions of TSP and PHP. (c) Infrared mean spectra of cystine derivatives in DH after treatment with different proportions of PBP and PHP.

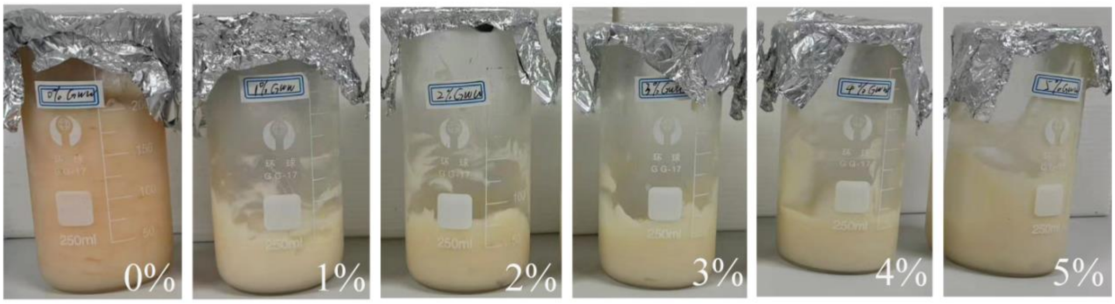

Figure S5. Emulsion samples made by adding different concentrations of TSP-PHP-PBP.

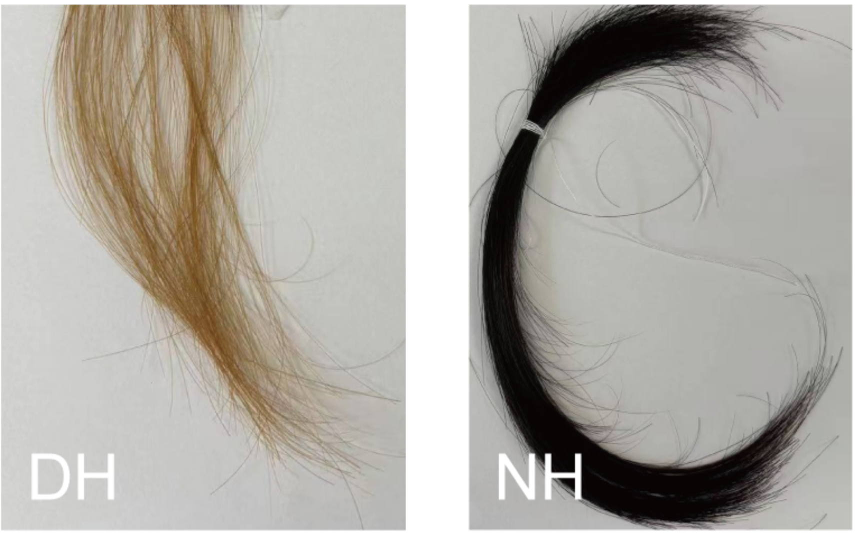

Figure S6. DH and NH samples.

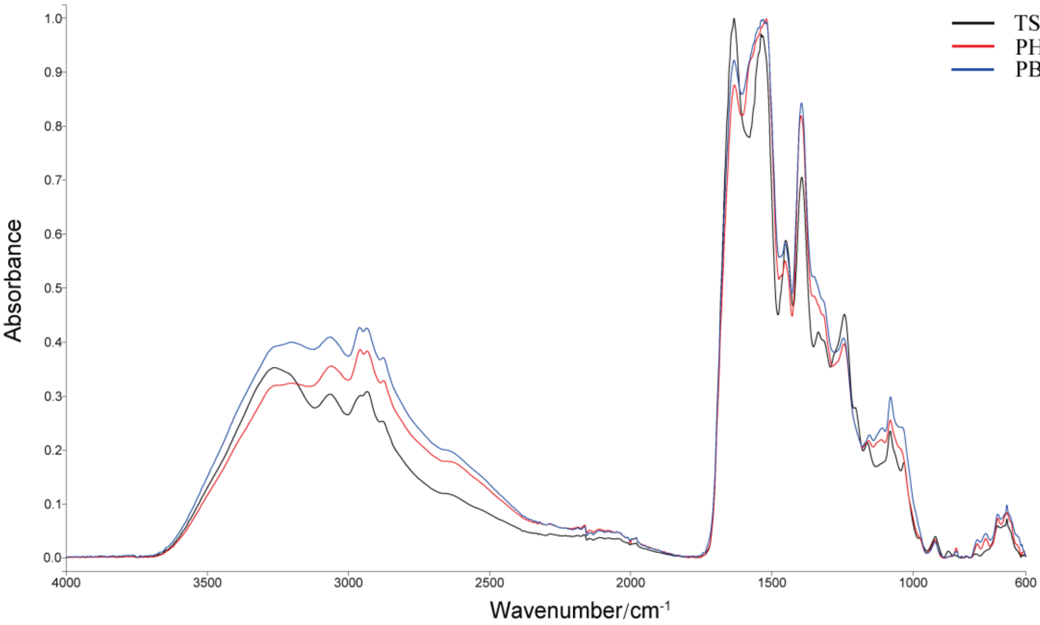

Figure S7. Infrared spectra of TSP, PBP, and PHP.

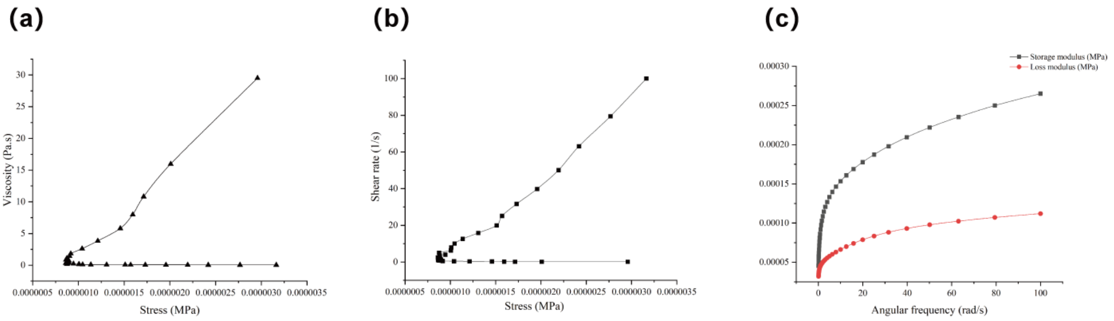

Figure S8. Rheological analysis of materials in CHFRE (a)The Effect of Shear Rate on the Viscosity of CHFRE;(b)The Effect of Shear Rate on the Stress of CHFRE;(c)The Effect of Frequency on the Storage Modulus (G') and Loss Modulus (G'') of CHFRE.

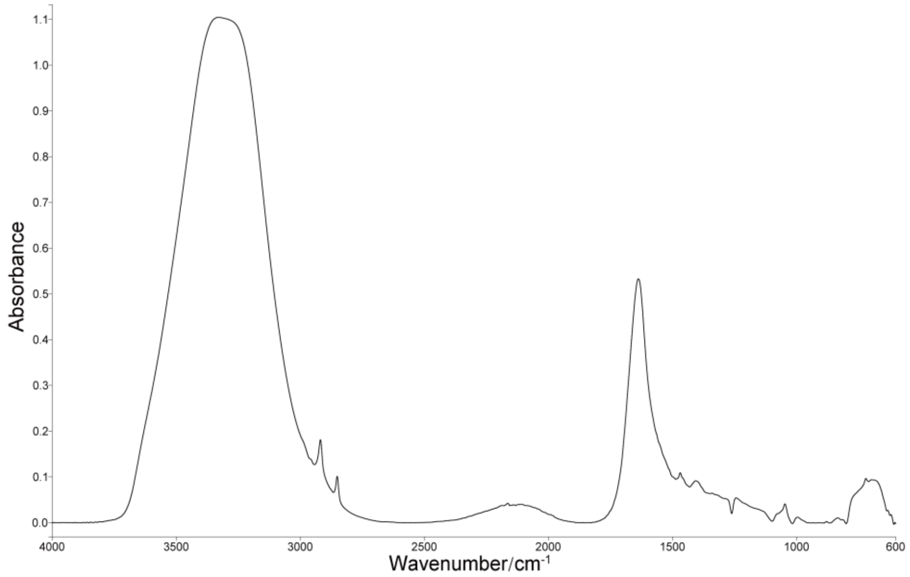

Figure S9. Infrared spectra of CHFRE(5% TSP-PBP-PHP).

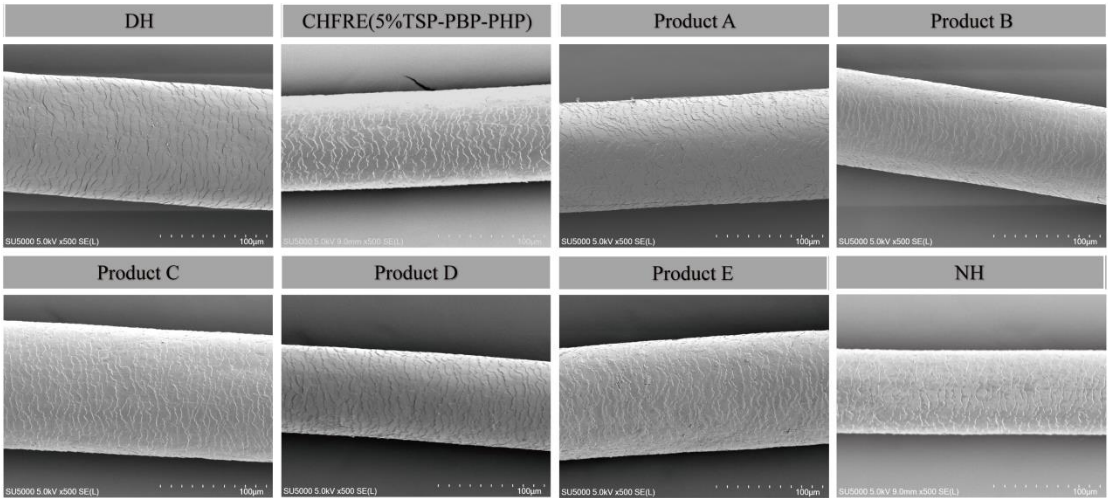

Figure S10. SEM images of DH and NH by 500×after hair care treatment with CHFRE(5% TSP-PBP-PHP) and Commercial Product (A,B,C,D,E).

Table

Table S1. Box-Behnken design results of α-conformation and transmittance.

	TSP numbers	PBP numbers	PHP numbers	α-conformation%	Transmittance %
1	3	6	7	27.89	47.71
2	9	9	4	32.57	52.03
3	6	9	7	28.98	47.78
4	6	6	1	32.78	49.90
5	9	6	7	31.56	49.25
6	3	6	1	26.13	46.73
7	9	3	4	33.19	51.68
8	6	6	4	30.21	50.54
9	6	6	4	29.01	50.82
10	3	3	4	27.10	48.47

11	6	6	4	29.16	50.18
12	6	6	4	30.25	50.25
13	6	6	4	29.76	50.44
14	6	3	7	28.95	49.32
15	6	3	1	27.58	47.76
16	6	9	1	26.98	48.94
17	3	9	4	27.19	50.50

Table S2. Analysis of variance and coefficient significance test of α-conformation regression model.

Source of variance	Square sum	Degrees of freedom	Mean square	F-value	P-value	Significance
Model	70.11	9	7.79	22.63	0.0002	significant
A-TSP	56.13	1	56.13	163.05	< 0.0001	
B-PBP	0.3612	1	0.3612	1.05	0.3397	
C-PHP	1.91	1	1.91	5.55	0.0506	
AB	0.0030	1	0.0030	0.0088	0.9279	
AC	2.22	1	2.22	6.45	0.0387	
BC	0.0992	1	0.0992	0.2883	0.6080	
A^2	4.01	1	4.01	11.62	0.0112	
B^2	1.02	1	1.02	2.95	0.1293	
C^2	4.77	1	4.77	13.85	0.0074	
Residual	2.41	7	0.3442			
Lack of Fit	1.08	3	0.3594	1.08	0.4527	not significant
Pure Error	1.33	4	0.3329			
Cor Total	72.52	16				

Table S3. Analysis of variance and coefficient significance test of transmittance regression model.

Source of variance	Square sum	Degrees of freedom	Mean square	F-value	P-value	Significance
Model	34.14	9	3.79	22.18	0.0002	significant
A-TSP	11.16	1	11.16	65.27	< 0.0001	
B-PBP	0.5101	1	0.5101	2.98	0.1278	
C-PHP	0.0666	1	0.0666	0.3895	0.5523	
AB	0.7056	1	0.7056	4.13	0.0818	
AC	0.6642	1	0.6642	3.88	0.0894	
BC	1.85	1	1.85	10.82	0.0133	
A^2	0.031	1	0.031	0.181	0.6833	
B^2	0.0805	1	0.0805	0.4706	0.5148	
C^2	19.18	1	19.18	112.15	< 0.0001	
Residual	1.2	7	0.171			
Lack of Fit	0.9392	3	0.3131	4.86	0.0805	not significant
Pure Error	0.2579	4	0.0645			
Cor Total	35.34	16				

Table S4. Amino acid content of TSP、PBP and PHP(g/100g).

Amino acid types	PHP	PBP	TSP
Asp	9.31±0.34	7.24±0.17	5.98±0.15

Thr	4.44±0.19	3.88±0.06	2.45±0.06
Ser	4.22±0.21	3.14±0.08	3.88±0.04
Glu	14.67±0.71	13.23±0.00	9.53±0.17
Gly	8.46±0.12	7.58±0.06	22.67±0.23
Ala*	6.89±0.09	6.19±0.00	10.11±0.01
Cys	0.29±0.17	0.09±0.02	0.05±0.00
Val*	4.41±0.11	3.88±0.02	2.52±0.09
Met*	2.72±0.01	2.22±0.17	0.52±0.02
Ile*	0.03±0.00	3.59±0.09	1.27±0.01
Leu*	6.57±0.26	6.62±0.32	3.05±0.04
Tyr	2.76±0.38	2.95±0.67	1.33±0.14
Phe*	4.13±0.65	3.66±0.9	3.72±0.11
Lys	7.60±0.16	4.01±0.04	4.17±0.15
His	2.28±0.31	2.12±0.03	1.34±0.23
Arg	6.81±0.31	1.44±0.01	8.07±0.22
Pro*	5.20±0.09	4.62±0.15	11.28±0.44
Hyp	0.01±0.00	0.01±0.00	4.29± 0.17
Total amino acids	29.92	30.77	32.48
Hydrophobic amino acid	90.76	76.45	94.05

Note: * are hydrophobic amino acids.(Based on the Previous Data from the Research Group.).

Table S5. Comparison of Repair Indices Between CHFRE and Commercial Product (A, B, C, D, E).

	**DH**	**CHFRE (5% TSP-PBP-PHP)**	**Product A**	**Product B**	**Product C**	**Product D**	**Product E**	**NH**
α-conformation (%)	27.39	33.33	32.35	31.71	28.85	28.31	35.08	33.91
⊿Hd (J/g)	5.707	7.330	7.980	6.450	5.930	5.660	11.230	10.460
Transmittance of S=O at 1042cm-1 (%)	43.23	52.31	54.63	47.20	47.25	22.35	52.94	53.16
Total amino acids(g/100g)	50.07	66.84	59.83	65.38	68.96	55.98	67.31	70.88
Hydrophobic amino acid(g/100g)	18.26	23.63	14.79	17.61	19.16	15.70	18.00	21.16
Antioxidant (μg/mL)	-	12.75	6.84	5.62	12.46	9.21	10.26	-
